# Sarcoma cell-specific radiation sensitization by titanate scrolled nanosheets: insights from physicochemical analysis and transcriptomic profiling

**DOI:** 10.1038/s41598-024-53847-x

**Published:** 2024-02-08

**Authors:** Pierre Beaudier, Florent Vilotte, Marina Simon, Giovanna Muggiolu, Quentin Le Trequesser, Guillaume Devès, Laurent Plawinski, Antoine Mikael, Jérôme Caron, Guy Kantor, Denis Dupuy, Marie-Hélène Delville, Philippe Barberet, Hervé Seznec

**Affiliations:** 1 UMR 5797, LP2I Bordeaux, CNRS, University of Bordeaux, 33170 Gradignan, France; 2 U1212, IECB, INSERM, University of Bordeaux, 33607 Pessac, France; 3 UMR 5026, ICMCB, Bordeaux INP, CNRS, University of Bordeaux, 33608 Pessac, France; 4 Radiation Oncology Unit, Institut Bergonié, 33076 Bordeaux, France

**Keywords:** Nanotechnology in cancer, Sarcoma

## Abstract

This study aimed to explore the potential of metal oxides such as Titanate Scrolled Nanosheets (TNs) in improving the radiosensitivity of sarcoma cell lines. Enhancing the response of cancer cells to radiation therapy is crucial, and one promising approach involves utilizing metal oxide nanoparticles. We focused on the impact of exposing two human sarcoma cell lines to both TNs and ionizing radiation (IR). Our research was prompted by previous in vitro toxicity assessments, revealing a correlation between TNs' toxicity and alterations in intracellular calcium homeostasis. A hydrothermal process using titanium dioxide powder in an alkaline solution produced the TNs. Our study quantified the intracellular content of TNs and analyzed their impact on radiation-induced responses. This assessment encompassed PIXE analysis, cell proliferation, and transcriptomic analysis. We observed that sarcoma cells internalized TNs, causing alterations in intracellular calcium homeostasis. We also found that irradiation influence intracellular calcium levels. Transcriptomic analysis revealed marked disparities in the gene expression patterns between the two sarcoma cell lines, suggesting a potential cell-line-dependent nano-sensitization to IR. These results significantly advance our comprehension of the interplay between TNs, IR, and cancer cells, promising potential enhancement of radiation therapy efficiency.

## Introduction

Radiation therapy (RT) is a pivotal cancer treatment used alongside surgery or chemotherapy, administered either before (neo-adjuvant) or after (adjuvant) treatment, to enhance its positive effects^1,2^. RT employs high-energy ionizing radiation (IR) to inhibit tumor growth. IR interacts with matter in two steps: (i) a physicochemical step involving atom excitation, ionization and water radiolysis, and (ii) a biological step, resulting in macromolecular alterations and cellular lesions that activate signaling pathways. External beam RT has the inherent potential to adversely affect healthy cells by inducing damage to their DNA or triggering cell death. The affected cells can undergo different outcomes such as recovery and return to normal function, cell death, or impaired repair, leading to DNA mutations and potential cancer development. For example, studies have shown that patients undergoing an RT treatment can face radiation-related risks, including cardiovascular complications, radiation pneumonitis, development of secondary cancers, and lymphedema^3–8^. The efficacy of RT hinges on delivering an effective dose to the tumor, with higher doses leading to better control, known as “*dose escalation*”. If the delivered RT dose fails to eliminate all cancer cells, there is a risk of future regrowth^9,10^. However, increasing the dose in the tumor tissue also affects the surrounding healthy tissue, leading to side effects that hinder treatment continuation. Therefore, ongoing developments in RT aim to refine the differential impact on tumors and healthy tissues. They encompass the exploration of innovative irradiation techniques based on ballistics and precise beam delivery alongside the exploration of the use of charged particles^11–15^.

Furthermore, research efforts concern new radio-sensitizing, radio-enhancing, or radioprotective agents against IR^16–19^. Among these, nanoparticles (NPs) have attracted significant attention in medicine due to their potential applications in drug delivery, contrast-enhanced imaging, and radio-sensitizing or radio-enhancement. The primary mechanism behind the ability of NPs to act as radiosensitizers relies on the increased absorption of X-rays, leading to the emission of secondary electrons and fluorescence photons^20^. This process involves photoelectric effect, Compton scattering, and pair production. At low energies (below 100 keV), the photoelectric effect governs the photon attenuation, while the Compton effect becomes the dominant process for energies between 100 keV and 10 MeV, and pair production dominates above 10 MeV^21,22^. Since clinical practice typically uses energies between 0.3 and 20 MeV, the Compton effect is the main contributor to energy deposition with a Compton cross-section proportional to Z. Thus, high atomic number elements received priority for radio-enhancement, with gold (Z = 79) widely studied for its biocompatibility and ability to enhance radiation therapy^23–26^. Other elements such as bismuth (Z = 83), platinum (Z = 78), and gadolinium (Z = 64) have also attracted interest as potential radiosensitizers^27^.

Gold NPs combined with X-rays improved survival rates in mice with mammary carcinomas^25^. Bismuth oxide NPs and bismuth selenide nanoplates exhibited dose enhancements both in vitro and in vivo^28–30^. Platinum NPs amplified gamma ray effects by over 40%, even in the highly radioresistant organism *D. radiodurans*^31^. Iron oxide NPs increased the radiotherapy effectiveness and the concentration of reactive oxygen species (ROS) in prostate carcinoma cells^32,33^. Gadolinium-based NPs, such as AGuIX, an ultrasmall formulation of polysiloxane and gadolinium chelates, proved in vitro efficacy on various cell lines, including a radioresistant head and neck squamous cell carcinoma^34,35^. The in vivo enhancement radiation sensitivity has also been demonstrated in glioblastoma, brain metastases, melanoma, pancreatic cancer, liver cancer, chondrosarcoma, head and neck cancer, and lung cancer^34^. Crystalline hafnium oxide NPs (Z = 72), referred to as NBTXR3, displayed benefits in survival, tumor growth delay, and local control when combined with radiation therapy (RT) in both mesenchymal and epithelial human tumor xenografts^36^. NBTXR3 gave promising results on sarcoma treatment in phase I and phase II–III of clinical trials^37^. Despite an uncertain mechanism of action, the phase II–III trial revealed a higher rate of pathological complete response in the NBTXR3 group compared to radiotherapy alone. The approval of NBTXR3 as a pioneering radiation enhancer for sarcoma treatment provided the potential to facilitate surgical resection. It can now serve as a model for enhancing RT in other solid tumors^38^.

In addition to the physical interactions mentioned above, NPs have the potential to induce a variety of biological effects. These responses include the generation of ROS, DNA damage, and changes in cellular metabolism^39^. Studies indicate that the NPs size influences ROS production and DNA damage, with high surface area to volume ratios leading to increased effects^40,41^. With IR, NPs can cause cell cycle arrest at the G2/M transition, leading to apoptosis^42–44^. Nanodiamonds also showed cell cycle arrest in G1/S followed by senescence^45^. Ghita M. et al*.,* using soft X-ray microbeams (carbon K-shell, 278 eV) to achieve selective cytoplasmic and nuclear irradiation, demonstrated that gold NPs could induce DNA damage and mitochondrial depolarization^46,47^. Studies discovered potent radiosensitization effects in vitro with minimal NP quantities, suggesting the influence of factors beyond NP dosage^40,48,49^. Oxidative stress has emerged as a pivotal player, with gold NPs inhibiting redox regulators such as thioredoxin reductase and glutathione reductase in both cancer and normal cells^48,50^. Some authors suggested the catalytic nature of the NPs, as radiolytic reactants present on their surface might impact their radio-enhancing abilities^51^.

Titanium dioxide NPs (TiO_2_, Z = 22) are versatile, finding use in medical applications like disinfection, dopamine detection^52,53^, bone growth enhancement^54^, and dental composites^55^. Their cytotoxicity depends on factors like morphology, chemical composition, and functionalization^56–58^. The NP shape also affects their biodistribution since we and others showed that elongated organic NPs are more efficiently taken up by cells than spherical ones^56,59,60^. TiO_2_ NPs can induce ROS generation^61–63^; they are here explored to optimize RT through in vitro testing. In particular, titanate nanotubes containing water, oxygen, and hydroxides can escalate free radical production when exposed to IR. This interaction increases the production of free radicals such as OH^⋅^, H^⋅^, and HO_2_^⋅^, known as radiosensitizers^44,64^.

In the present study, our objective is to comprehensively assess how exposure to the combination of TNs and IR impacts cellular homeostasis, cell proliferation, and gene expression.

## Results

### Synthesis and physicochemical properties of titanate scrolled nanosheets (TNs)

**Figure 1:**
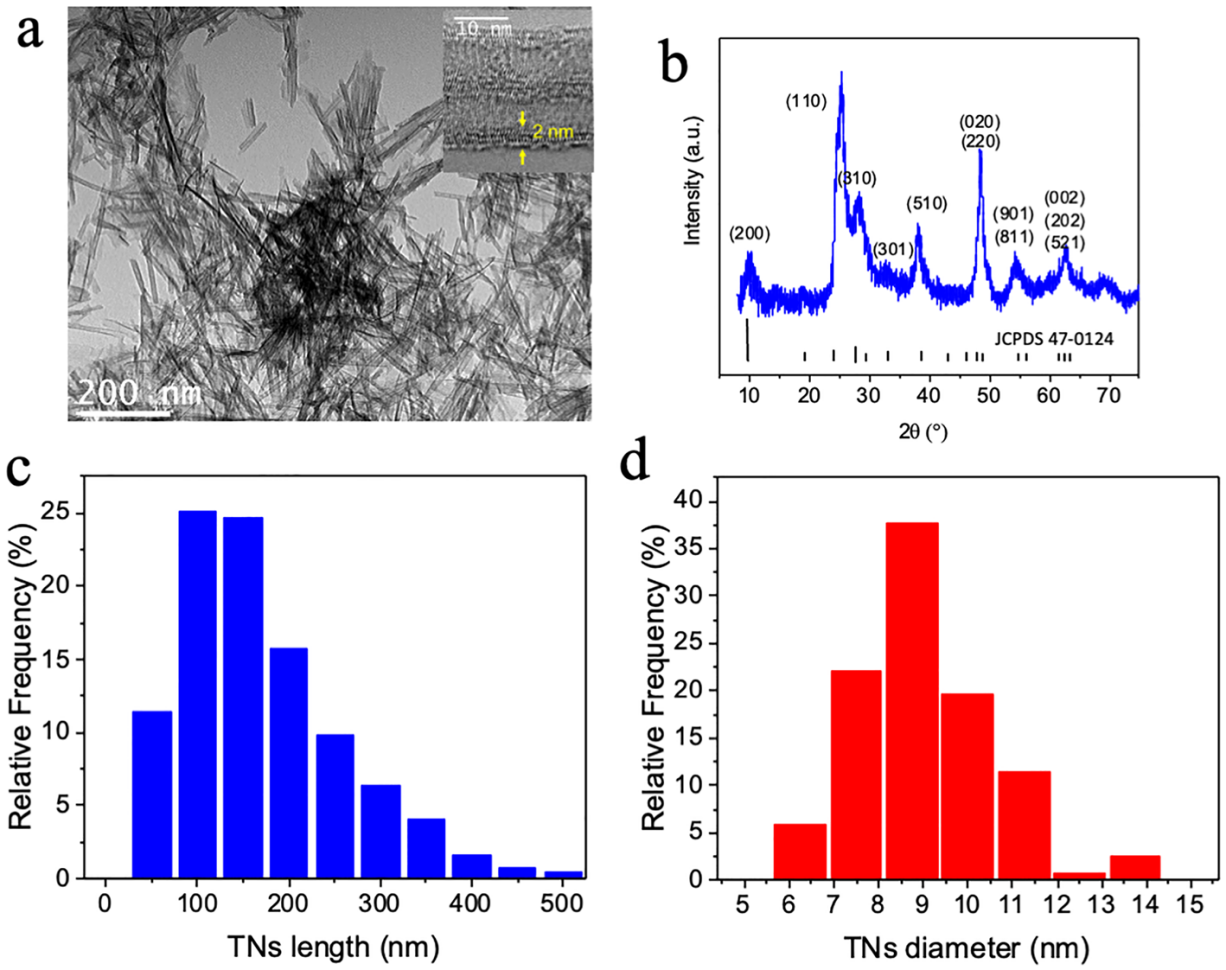
Titanate scrolled nanosheets (TNs). (a) Transmission electron microscopy images of synthesized Titanate scrolled nanosheets (Inset: high-resolution image showing the TN wall thickness. (**b**) XRD pattern of the TNs in (**a**). Histogram of the size distribution of the TNs measured on at least 300 NTs: (**c**) length in nm (**d**) diameter in nm.

We synthesized titanate scrolled nanosheets (TNs) from AEROXIDE P25 NPs, which consist of 75% anatase and 25% rutile^65–67^. The alkaline-based hydrothermal synthesis developed by Kasuga et al*.* rapidly turned out to be the main non-templated method for the production of TNs^68^ or layered titanate nanomaterials^65–67^. P25 TiO_2_ is mixed into a 10 M NaOH aqueous solution, which is then submitted to hydrothermal treatment at 130 °C for 20 h, in a Teflon-lined autoclave. The product is washed with a 0.1N HNO_3_ solution, to reach a pH close to 7 at which point the slurry is filtered. TNs are either dried in air for analysis or kept in water for further use. The Transmission Electron Microscopy (TEM) image and XRD pattern of such synthesized TNs are shown in Fig. 1 and further detailed characteristics can be found in Simon et al*.*^56^ and Bai et al*.*^69^.

The elongated nanoobjects are crystalline and consist of a thin curved structure of several layers of a single nanosheet wrapped on itself, along the *c* axis, leading to a nanotube with an average external diameter of about 9 ± 1.6 nm with a multiwall thickness of about 2 nm and a length of several hundred nanometers^68^. The transparency of the objects in their central part indicates a lower density and confirms their tubular morphology (Fig. 1a). The interlayer spacing of these nanotubes is 0.7 nm, close to reported values in the literature^6,70–73^. The average tube size is 172 ± 94 nm, indicating a high degree of dispersity. However, their diameter varies only slightly. The XR diffractogram of these nanosheets corresponds to a hydrogen titanate phase and the peaks have been indexed according to the H_2_Ti_2_O_5_⋅H_2_O phase (JCPDS No 47-0124), which is very close to the structure of nanosheets found in the literature^74,75^ (Fig. 1b). The crystal structure of these TNs is still a matter of debate. Several structures have been proposed for these objects, such as the monoclinic lattice H_2_Ti_3_O_7_, the lepidocrocite H_0.7_Ti_1.825_◊_0.175_O_4,_ and finally the orthorhombic phase H_2_Ti_2_O_5_^67,75–81^_._ However, the community as a whole is unanimous on obtaining a protonated form of titanium oxide or hydrogen titanate that can be written as H_2m_Ti_n_O_2n+m_^82^. Throughout the synthesis process, we kept the TNs in solution to prevent aggregation caused by drying. Despite their negative surface charge at physiological pH 7.4, TNs flocculated and quickly agglomerated after sonication even if easily re-dispersible. A stock solution with a concentration of 1 mg.ml^-1^ is used to achieve a cell exposure dose of 2 µg.cm^−2^^56^.

### Cell proliferation assay reveals differences in sarcoma cell lines based on exposure conditions: impact of TNs and ionizing radiation (IR)

#### Choice of sarcoma cells

Sarcomas represent a heterogeneous group of rare tumors, accounting for approximately 1% of adult cancers with more than 50 histological subtypes^83^. They are derived from mesenchymal tissue including bones, muscles, cartilage, and other connective tissues. Sarcoma etiology is unknown, but external RT is a well-established risk factor for soft tissue sarcoma. Indeed, sarcomas belong to the type of cancer that can be radiation-induced, and they present a higher resistance to conventional RT. We used two sarcoma cell lines^83^. The first, named IB115, was obtained from a dedifferentiated liposarcoma of a para-testicular tumor after surgery. IB115 has a simple genetic profile based on many known limited amplifications of the MDM2/CDK4 genes on chromosome 12q15 (Supplementary Information Fig. S1). The second, named IB106, is an unclassified sarcoma from the paravertebral mass with heterogenic pleomorphic cells with a complex genetic. Genomic stability was assessed over 50 passages via Comparative Genomic Hybridization (CGH). The CGH profiles remained unchanged, as evidenced by the overlapping CGH profiles in passages 30 and 50. This highlights their remarkable genomic stability and reinforces the interest in using these cell lines for in vitro studies^83^ (Supplementary Information, Fig. S2).

#### Sarcoma cell exposure to NTs alone

**Figure 2:**
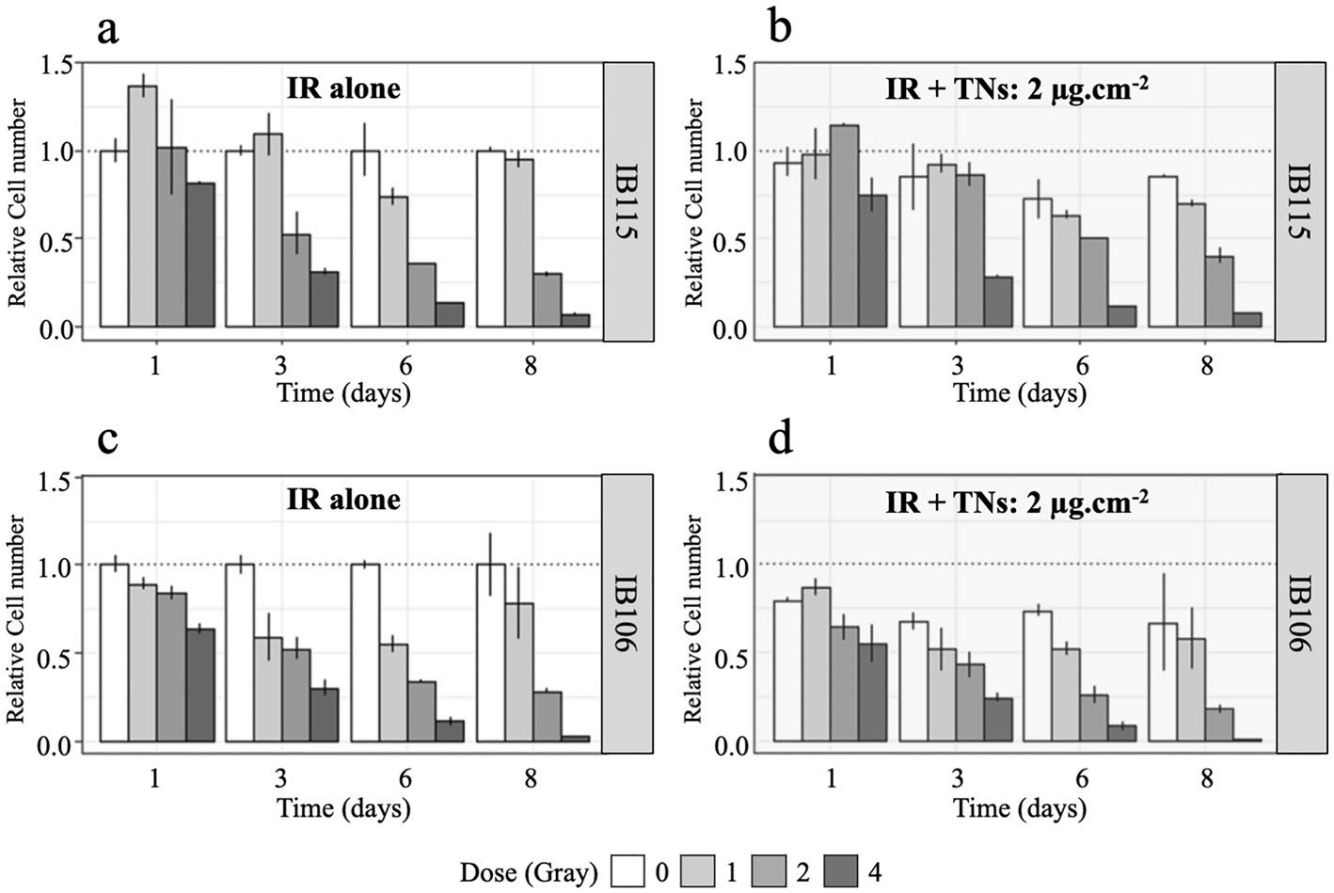
Cell proliferation assay on sarcoma cells under different exposure conditions. The relative number of cells *per* well *per* day was measured following an initial exposure to TNs for 20h at a dose of 2 µg.cm^-2^, followed by a second exposure by irradiation at different doses expressed in Gy (mean value ± standard deviation, n = 2). The irradiation doses ranged from 1 to 4 Gy, depicted in grayscale from white (control) to dark gray (4 Gy). Cell proliferation was evaluated over an 8-day post-irradiation. (**a**) IB115: irradiation without TNs. (**b**) IB115: irradiation in the presence of internalized TNs. (**c**) IB106: irradiation without TNs. (**d**) IB106: irradiation in the presence of internalized TNs.

The toxicity of TNs has been assessed by cell proliferation assay. We based our choice on previous studies, which encompassed various cell lines such as primary keratinocytes, endothelial cells, and commercially available cancerous cell lines such as HeLa and HTB96 U2OS^56,84^. Figure 2 shows the results of a cell proliferation assay performed on IB106 and IB115 under different exposure conditions. One day after exposure to TNs, IB115 and IB106 displayed a decrease in cell proliferation of approximately 30% for IB106 and about 10% for IB115 at 0 Gy (Fig. 2b, d, white boxes). IB106 consistently exhibited higher sensitivity to TNs, as evidenced by the significant decrease in proliferation throughout the experiment. In contrast, IB115 showed a lower susceptibility to TNs exposure, with a weaker impact on the proliferation 24 h after the exposure and subsequent recovery of proliferative activity over several days (Fig. 2b, white boxes).

#### Sarcoma cell exposure to IR alone

The effects of IR on cell proliferation are illustrated in Fig. 2a, c, which show the relative cell number *per* well *per* day during 8 days for irradiation doses ranging from 0 to 4 Gy. Notable differences in plating efficiency were observed between IB115 and IB106. One day after irradiation, IB115 exhibited a higher cell number in the irradiated 1 and 2 Gy conditions, suggesting a potential promotion of cell plating by irradiation (Fig. 2a).

Conversely, IB106 displayed a marked decrease in the cell number. These results suggest that IB106 is more sensitive to radiation than IB115. The proliferation rate of the two cell lines decreased upon irradiation, IB106 showing a rapid and significant decline with increasing dose (Fig. 2c), whereas IB115 demonstrated a progressive and delayed decrease, mainly at 2 and 4 Gy (Fig. 2a). A tendency to go back to a normal proliferation rate appeared after 6 days at a dose of 1 Gy, and both cell lines had a maximum decrease in proliferation at 6 days after irradiation (Fig. 2a, c).

### Sarcoma cell exposure to IR in the presence of TNs once internalized in cells

IB115 and IB106 were first exposed to TNs for 20 h and to the different IR doses. Both cell lines exhibited alterations in their proliferation compared to the control (Fig. 2b, d, white column). After TNs internalization due to exposure to TNs at a dose of 2 µg.cm^−2^ for 20 h, the cells were subsequently irradiated at doses ranging from 1 to 4 Gy (6 MeV photon beams). The combined effect of internalized TNs and IR on cell proliferation induces a significant decrease in the cell number for both cell lines (Fig. 2b, d). This decrease reaches a maximum on day 6 for all the doses. Significantly, there is a clear contrast in sensitivity between the two cell lines. IB106 displays a slightly enhanced response to both TNs and IR, particularly at the 1 Gy dose. On days 6 and 8 in the absence of TNs, cells exposed to a radiation dose of 1 Gy displayed an increase in cell number. This indicated that their proliferation was not definitively impaired. However, in the presence of TNs, this increase in cell number was not observed, suggesting a potential interaction between TNs and the cellular response to radiation.

### Effect of TNs and IR on sarcoma intracellular calcium content

**Figure 3:**
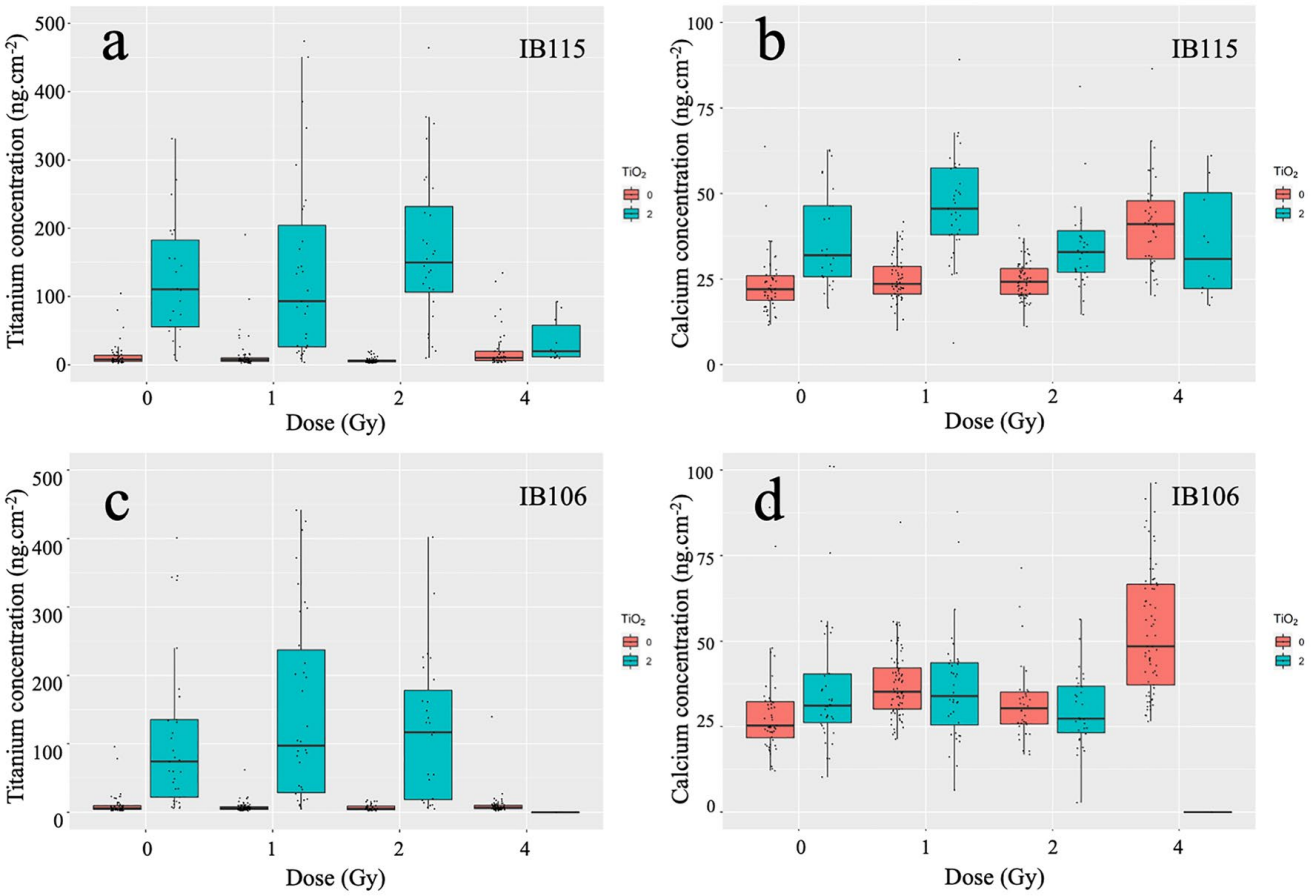
TNs, irradiation, and calcium homeostasis. Quantitative analysis of the total intracellular titanium and calcium contents by micro-PIXE in sarcoma cell lines in the different tested experimental conditions, i.e., in the presence (or not) of 2 µg.cm^−2^ of TNs and combination (or not) of irradiation (0, 1, 2 and 4 Gy). Quantitative analysis was performed in the absence of TNs (red) or in the presence of TNs (blue). Sarcoma cell lines are depicted in rows (IB115-top; IB106-down). (**a,c**) Titanium content is expressed in ng.cm^−2^ for IB115 and IB106, respectively. (**b,d**) Calcium content is expressed in ng.cm^−2^ for IB115 and IB106, respectively.

We investigated both the intracellular content of titanium (Ti) and its influence on intracellular calcium concentration using micro-PIXE (Particle-Induced X-ray Emission) (Fig. 3).

The results depicted in Fig. 3 focus on the quantification at the single-cell level of titanium uptake (Fig. 3a, c) and calcium uptake (Fig. 3b, d), expressed as normalized values in ng.cm^-2^. First, the analysis revealed the presence of Titanium (Ti) with no notable difference in quantity and distribution between IB115 and IB106. No differences in the composition and distribution of chemical elements such as Na, Mg, Cl, P, K, and S were noticed across the various conditions (*data not shown*). Nevertheless, intracellular titanium content in both cell populations exhibited a marked heterogeneity, ranging from 50 to 250 ng.cm^−2^, as documented in previous studies^84^.

The intracellular calcium levels were also modified, considering not only the impact of both IR (Fig. 3b, d, red boxes, 1–4 Gy) and TNs (Fig. 3b, d, green box, 0 Gy ) acting separately but also the combined effect of these 2 exposures (Fig. 3b, d, green boxes, 1–4 Gy). Our results revealed a substantial presence of intracellular calcium in the two cell lines under baseline conditions, without TNs nor irradiation (ranging from 22 ± 5 to 26 ± 7 ng cm^−2^ in controls), with no significant difference between the cell lines. Exposure to TNs without irradiation significantly raised intracellular calcium content in IB115 (32 ± 14 ng.cm^−2^), whereas IB106 experienced no significant change (calcium content increase, 31 ± 9 ng.cm^−2^). When considering IB115 under irradiation alone, no difference in calcium content was observed between controls and those irradiated at doses of 1 and 2 Gy. A difference was only observed at a dose of 4 Gy. Moreover, the presence of both TNs and irradiation induced a marked increase in the calcium content within the same range as that observed for 4 Gy irradiation alone. These observations suggest that (i) irradiation has no impact on the calcium homeostasis in IB115, unlike TNs; (ii) IB115 cells exhibit resistance to irradiation at doses below 4 Gy; and (iii) the presence of TNs alone is sufficient to induce a similar effect as a 4-Gy irradiation.

In IB106, except for the condition at 4 Gy with a notable increase in calcium content observed, Fig. 3 illustrates that both irradiation and TNs resulted in a moderate increase in intracellular calcium content. The facts that (i) the presence of TNs consistently correlated with an increase in intracellular calcium content, and (ii) the irradiation alone did not have a significant effect could indicate that TNs induce a maximum increase in intracellular calcium content, and irradiation does not further enhance this effect under any condition. Unlike IB115, the differences in calcium content increase in IB106 were relatively low (31 ± 9 ng.cm^−2^). However, it should be noted that at a dose of 4 Gy, cells exposed to both TNs and irradiation could not be analyzed due to extensive cell death. This observation further supports the argument that IB106 cells were indeed sensitive to both IR and TNs and that a minimum irradiation dose is mandatory.

The changes in the intracellular calcium concentration induced by the presence of both TNs and irradiation have a minimal effect on the cell proliferation capacity of IB115 while in contrast, they significantly impact the response capacity of IB106 to TNs.

### Transcriptomic analysis showed significant differential gene expression, supporting the hypothesis of TNs-induced sensitization to IR

**Figure 4:**
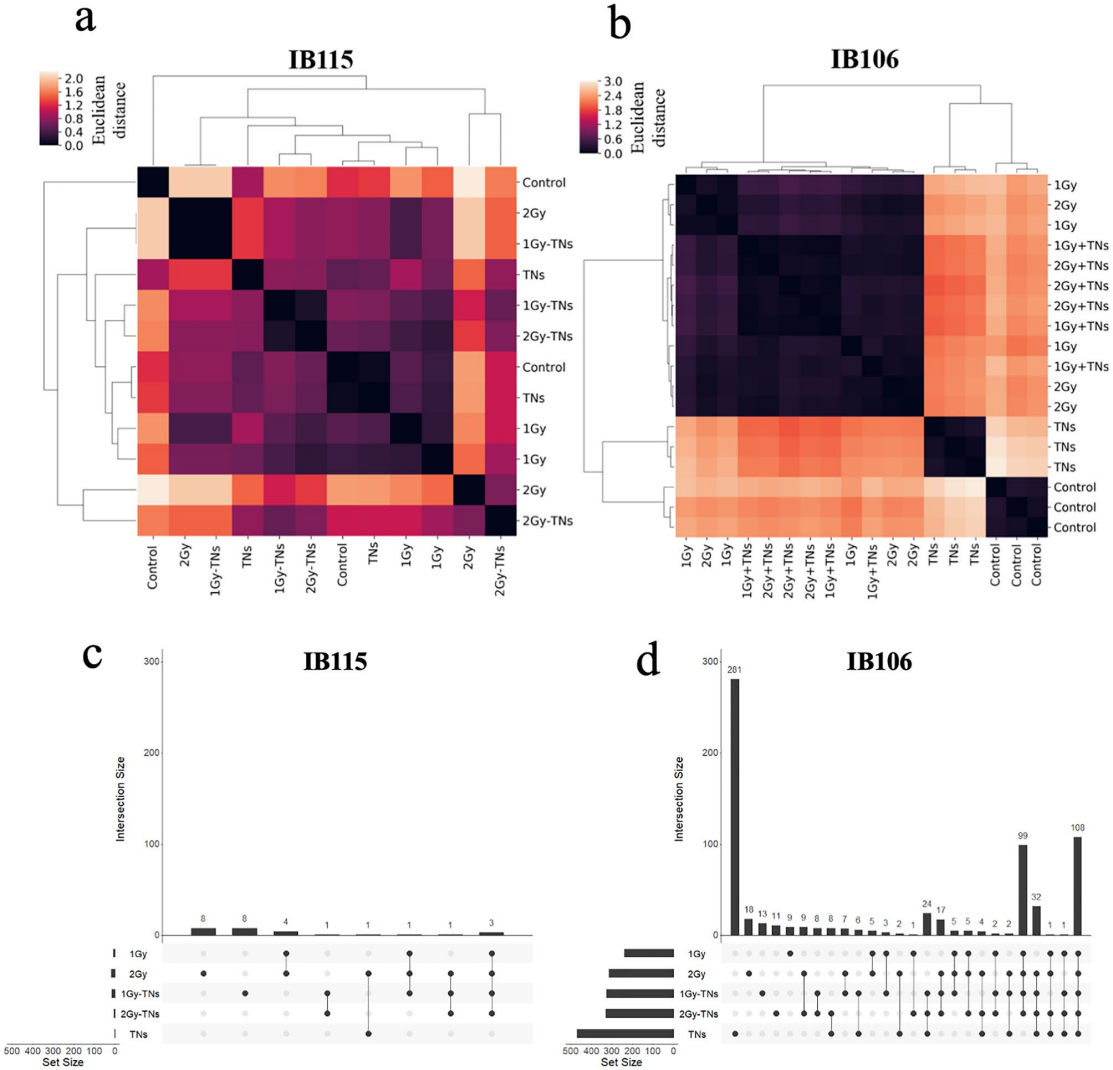
Cluster map of Euclidean distances extracted from the PCA plot and *upset* plots. (**a–c**) Cluster map of Euclidean distances extracted from the PCA plot (**a**) IB115, (**b**) IB106. Samples with low Euclidean distances (black) are more similar in expression than samples with high Euclidean distances (white). (**b–d**) *Upset plot* showing intersections of differentially expressed genes between experimental conditions. (**c**) IB115, (**d**) IB106. Horizontal line: experimental condition. Vertical line: intersection size between conditions marked with a black dot.

We investigated the involvement of some specific cellular mechanisms in responses to TNs and radiation exposures. We examined the influence of these conditions on all the transcriptomic responses in both cell lines. Transcription analysis provided insight into the physiological status of the cell lines under different stress conditions. We performed principal component analysis (PCA) on expressed genes to identify which gene expression could discriminate between exposure conditions (Supplementary Information Figs. S3a and S4a). PCA is a mathematical method used to reduce the dimensionality of complex data while retaining important information. During the process, new variables, Principal Components (PC), are built from genes with the most variation among all samples. These genes are ranked into multiple PCs (PC1, PC2, PC3, etc.), each describing a decreasing proportion of the variability in the data. While PCA does not provide precise information on transcriptomic expression profiles, it does offer an overview of the global distribution of each cell line according to the experimental conditions. It is, therefore, performed on the non-normalized expression matrix to capture the major expression trends that differentiate the experimental conditions (Fig. 4).

We observed significant differences between the cell lines. The IB106 cluster map clearly shows three groups of samples with similar expressions: controls, irradiated, and TNs only. The absence of a discernible structure for IB115 indicates that no particular group stands out, making it impossible to discriminate between samples from a specific experimental condition. Lower eigenvalues of PC1 (19%) and PC2 (16%) for IB115 than for IB106 (29% and 26%) indicate a reduced percentage of variance within the set of samples explained by these principal components. This suggests that in IB115, it is more challenging for the algorithm to identify distinct expression trends. For the PC1 and PC2 of each cell line, we extracted the 100 genes with the highest weight. PC1s from both cell lines had only 17 genes in common, while PC2s shared 73 genes. This tends to confirm that the overall expression trends identified by the PCA in the two cell lines do not involve the same genes overall; with less than half of the genes in the PC1 and PC2 components being in common between the two cell lines.

We then performed a differential expression analysis on all conditions and compared them to controls to compute differentially expressed (DE) genes (Fig. 4c, d). The observed difference in the number of DE genes aligns with the findings from PCA, indicating a clear transcriptomic response in IB106 (239 to 469 DE genes) and the absence of such a response in IB115 (1 to 18 DE genes). In IB106, TNs alone exhibited 469 DE genes, the highest amount found across all conditions (Fig. 4d and Supplementary Information Fig. S4b). Although we cannot quantify the exact level of response based solely on the number of DE genes, it is noteworthy that the combined action of TNs and IR results in a decrease in the number of DE genes (1 Gy-TNs: 327 genes, 2 Gy-TNs: 330 genes) compared to the exposure to TNs alone, although higher than irradiation alone (1Gy: 239 genes, 2 Gy: 314 genes). This observation suggests that the cellular response to irradiation has a more pronounced influence than the response to TNs in this particular cell line (Fig. 4d and Supplementary Information, Fig. S4b).

**Figure 5:**
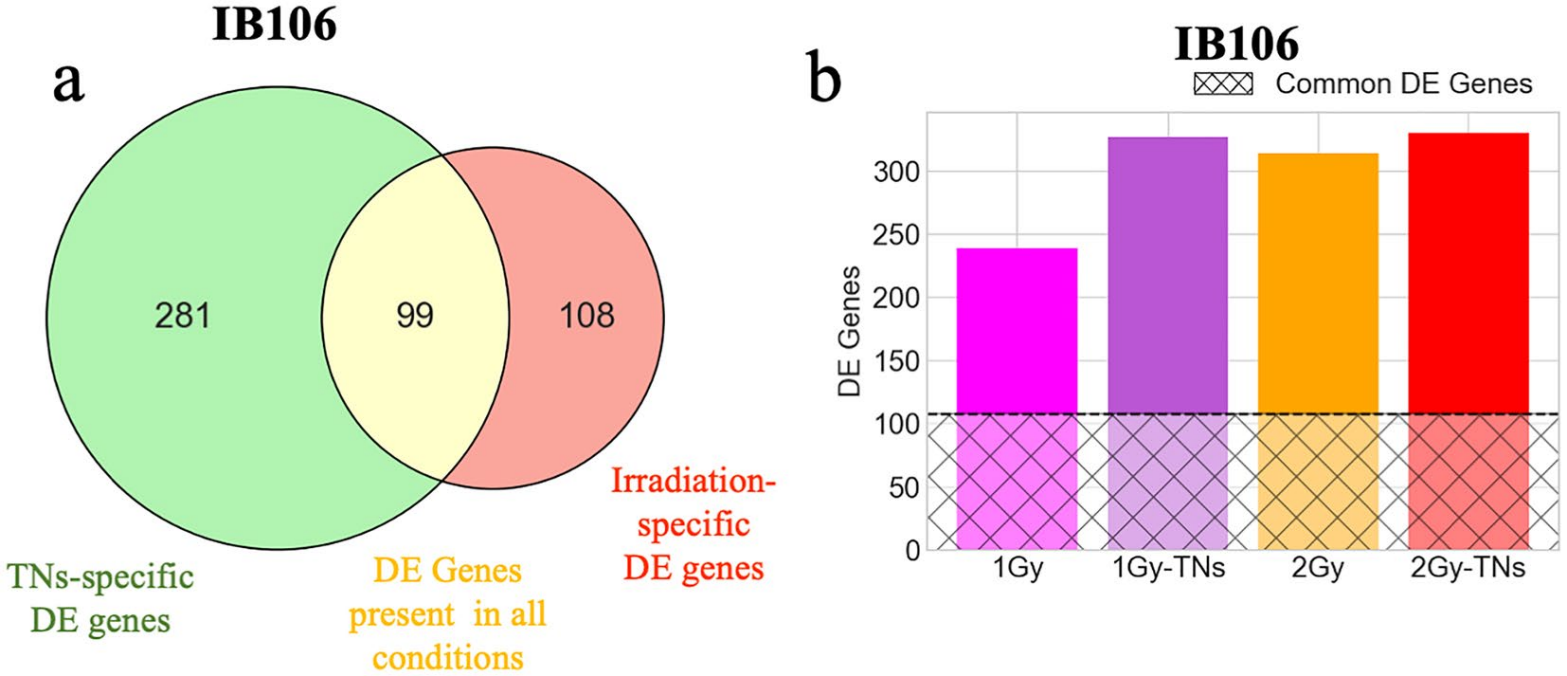
Differential gene expression in IB106. (**a**) *Venn* diagram of common and condition-specific DE genes in TNs and irradiation samples from the IB106 cell line. (**b**) Number of DE genes identified *per* irradiation condition from the IB106 cell line. The hatched area represents the amount of DE genes common to all irradiation conditions.

In the case of IB115, only 1 DE gene is identified under the TNs condition. In the irradiation conditions (1 Gy: 8 genes, 1Gy-TNs: 14 genes, 2 Gy: 18 genes, 2 Gy-TNs: 5 genes), with 3 genes being common to irradiation and irradiation + TNs conditions (Fig. 4c and Supplementary Information, Fig. S3b). As the cellular response to the different experimental conditions in IB115 was weak, we focused on the DE genes identified in IB106. We were particularly interested in the difference in response between the irradiation conditions and the TNs-only condition. The PCA already showed that not only did the cellular response to irradiation differ significantly from the response to TNs but also that the response to irradiation seemed to eclipse that to TNs, with irradiation + TNs conditions grouped with irradiated conditions without TNs. This observation is confirmed in the DE genes identified in the different conditions of the IB106 line (Fig. 5).

Three main groups of DE genes can thus be identified: (i) DE genes common to all experimental conditions (99 genes), which should therefore correspond to general stress pathways, (ii) DE genes specific to TNs-only conditions (281 genes), which should correspond to the specific response to TNs, and (iii) DE genes common to all irradiation conditions (108 genes), with or without TNs, which should correspond to the specific response to irradiation. Not all DE genes were found in these three groups. For example, irradiation-specific DE genes accounted for less than half of all DE genes found in each irradiation condition (Fig. 5b). Despite their proportion, these genes effectively represented the two distinct expression profiles identifiable in these data. The high number of DE genes specific to the TNs-only condition, and therefore absent in the irradiation + TNs conditions, confirms the PCA observation that the cellular response to irradiation supersedes the response to TNs.

In IB106, we observe a positive correlation between the number of DE genes and the number of impacted Gene Ontologies (GOs) (Supplementary Information, Fig. S4c). In contrast, the low number of DE genes in IB115 limits our ability to classify GOs as significantly impacted, with only a few found in the 1Gy-TNs and 2Gy conditions (Supplementary Information, Fig. S3c).

**Figure 6:**
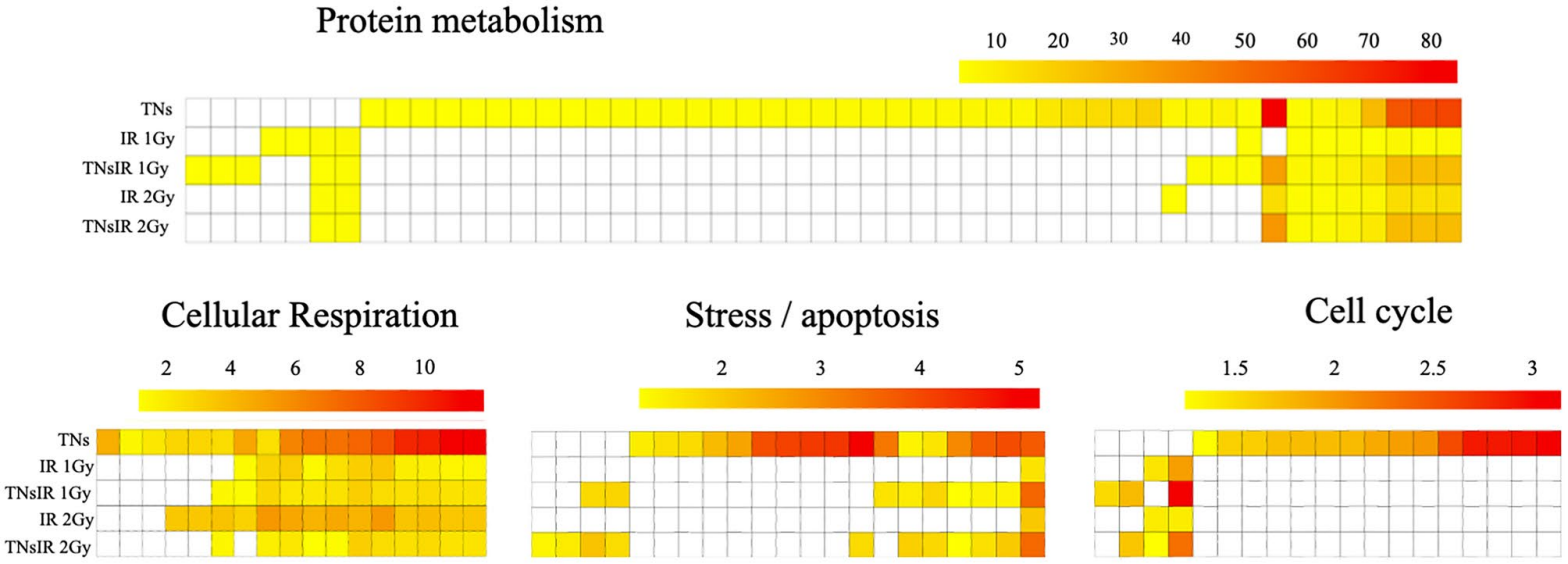
Heatmap of target genes ontologies (biological processes) significantly impacted in the IB106 line. Genes ontologies (biological processes) significantly impacted (adjusted *p-value* < 0.05) related to the four identified metabolic pathways (GO:Protein metabolism, GO:Cellular respiration, GO:Stress/apoptosis, GO:Cell cycle) and to the different experimental conditions. Each line corresponds to a Gene Ontology (column for protein metabolism). Color corresponds to the negative log of the *pvalue* from the gprofiler significance test (red: lower *p-values*, yellow: higher *p-values*). White cases mean the Gene Ontology is not impacted in the corresponding experimental condition.

Our analysis revealed four main relevant cellular pathways that impacted IB106: (i) protein metabolism, (ii) cell cycle, (iii) cellular respiration, and (iv) cell stress/death (Supplementary Information, Table S1–S4). As illustrated in Fig. 6, the majority of impacted ontologies undergo significant modification in response to the exposure to TNs. These suggest that TNs induced a global alteration of general metabolic pathways such as protein metabolism, cellular respiration, and cell cycle regulation. These observations have been partially described previously with the induction of the Endoplasmic Reticulum stress pathways (ER-Stress) and mitochondrial stress^56^. Nevertheless, these changes sign an unusual cellular status that could alter the radiation-induced response and thus promote the deleterious effects of IR.

## Discussion

Here, we focused on (i) synthesizing and characterizing titanate scrolled nanosheets (TNs) and (ii) assessing their effects in combination with ionizing radiation (IR) on two sarcoma cell lines. Micro-PIXE analysis confirmed that sarcoma cells internalize TNs as shown with TiO_2_ NPs by various cell types^5,8,60^. Quantification of intracellular titanium content revealed variation in cellular uptake, irrespective of their size, shape, and surface reactivity. Furthermore, Mirjolet et al*.* have provided evidence of TN internalization in glioblastoma cell lines^44^. Additionally, they showed that TNs were mainly located in the cytoplasm rather than in the nucleus. Since the primary effect of IR is DNA damage, it would be interesting to consider whether the localization of TNs facilitates or not the emergence of non-targeted effects of IR.

We then confirmed that the intracellular internalization of titanium (via TNs) did induce an alteration in the intracellular calcium content, as previously reported^56^. Interestingly, we did not observe any significant difference in the intracellular uptake of titanium (TNs) between the two cell lines. However, the presence of this intracellular titanium significantly influenced the intracellular calcium content, and this impact varied depending on the nature of the cell lines. Hence, we conclude that discrepancies in performed biological assessments, such as cell proliferation and transcriptomic analysis, could not be attributed solely to the variations in the intracellular TNs content within the studied cell populations. Instead, it seems that the alteration of intracellular calcium induced by the presence of intracellular titanium holds a central position in shaping calcium-dependent biological responses.

Our results revealed variations in cell proliferation, indicating the influence of TNs on both cell lines, with varying degrees of sensitivity; IB106 exhibiting the most pronounced response to TNs. Also, note that the intracellular content of titanium (TNs) is likely to decrease gradually within the cells as they proliferate and divide. Then, once TNs content has reached a minimal level, the cells regain their normal proliferation rate^56^. The fact that TNs induce a decrease in cell proliferation illustrates a potential fragilization of the cancer cells and supports the radiosensitization hypothesis, where TNs could enhance the effects of IR. The link between the intracellular content of TNs and the alteration in intracellular calcium homeostasis is a significant finding since it can influence various cellular and signaling mechanisms, including reactive oxygen species (ROS) production, mitochondrial metabolism, and inflammation. Of particular interest is the fact that the two cell lines, when exposed to IR, have a similar alteration in calcium homeostasis. TNs may reinforce this effect for the sarcoma cell lines studied. Since TNs and IR used separately had a detrimental effect on cancer cells, their combination could lead to an amplified disruption of calcium regulation within the cells, resulting in increased oxidative stress, inflammation, and potential damage to cellular structures. Our results highlight the complex interplay between TNs, IR, intracellular calcium homeostasis, and associated cellular responses.

The transcriptomic analysis, which provides valuable insights into the changes in gene expression that occur in response to the combination of TNs and IR, corroborates this hypothesis. We identified specific gene ontologies modulated by the interaction between TNs and IR. These include cellular respiration, apoptosis, cell cycle, and protein metabolism. All these pathways are known to be related to calcium homeostasis. The latter could be the expression of the ER-stress response as previously described in the context of TiO_2_ NPs toxicity evaluation^56^. Additionally, the distinct transcriptomic profiles observed in the two cell lines indicate that they respond differently to TNs and to the combination of TNs and IR. This helps us to understand the underlying mechanisms by which TNs enhance the effects of radiation and contribute to the observed cell sensitivity.

Indeed, in the case of IB115, the lack of significant induction in cell signaling indicates a reduced ability of these cells to respond to or sense the presence of TNs and the impacts of IR. As a result, the cellular response pathways associated with intracellular calcium homeostasis and related stress signaling in these cells are likely altered. This could result in a decreased sensitivity to the synergistic effects of TNs and IR, potentially contributing to cellular stress resistance. Understanding the factors influencing the resistance observed in IB115 not only provides insights into potential intervention targets but also sets the stage for developing strategies to enhance the sensitivity of cancer cells in the context of future therapy.

## Conclusion

Our results, taken together, highlight the importance of considering the impact of TNs on intracellular calcium homeostasis. They provide a basis for further research to understand the underlying mechanisms responsible for these effects in different cell types, including sarcoma cells. This detrimental alteration in intracellular calcium levels in the cytoplasm opens new possibilities to optimize radiotherapy using sensitization with metal oxide nanoparticles (TNs in particular). Furthermore, intracellular calcium content may serve as a bioindicator of nanotoxicity and may help researchers and clinicians evaluate the potential safety and effectiveness of TNs or metal oxide nanoparticles as radiosensitizers in combination with radiation therapy. Further research is needed to validate these findings, establish the optimal treatment conditions, and better understand the biological mechanisms involved, ultimately paving the way for potential clinical applications in the future. This approach includes evaluating the dose-dependent sensitivity of various cancer cell types to the combination of TNs and different types of ionizing radiation (Energy, Linear Energy Transfer, irradiation modality). Finally, the development and optimization of TNs delivery and the specific targeting of cancer cells are essential steps to master their potential use as radiosensitizers and to promote their clinical application in combination with radiotherapy. To further progress in this direction, researchers need to develop and validate three-dimensional (3D) multicellular biological models. They should have such a design to fit with experiments under conditions of combined exposure to titanium oxide nanomaterials and ionizing radiation. 3D multicellular models would, indeed, provide a more realistic representation of the tumor microenvironment when compared to traditional 2D cell cultures. Such a model would constitute a valuable platform for studying the efficacy, safety, and underlying mechanisms of TNs as radiosensitizers.

## Material and methods

### Synthesis and characterization of titanate-scrolled nanosheets

P25 nanoparticles (P25, AEROXIDE) were kindly provided by Degussa/Evonik and used as a precursor for the synthesis of titanate-scrolled nanosheets (TNs). TNs were produced via the hydrothermal process described by Kasuga et al*.*^67^. Briefly, 2 g of P25 were introduced in a 50 mL Teflon-lined autoclave with 28 mL of 10 M sodium hydroxide solution, sealed and heated at 130 °C for 20 h. The white precipitate was washed with nitric acid (0.1 M) and water for neutralization and identified as TNs. The obtained white powder was finally washed with deionized water. Synthesized TNs were kept in an aqueous solution avoiding aggregation issues. Mass concentrations were measured by drying a known volume of solution and weighing the extracted powder. Suspensions with a concentration of 1 mg.mL^−1^ were finally produced, sonicated, and kept in the dark. The physicochemical properties of TNs were characterized by standard techniques. Specifically, the primary size and morphological features of TNs were observed by TEM and high-resolution transmission electron microscopy (HRTEM) using a Hitachi H-7650 transmission electron microscope (TEM, 120 kV, Hitachi High-Tech Company, Japan), and a JEOL 2200 FS equipped with a field emission gun, operating at 200 kV and with a point resolution of 0.23 nm. HRTEM micrographs were acquired with a GatanUltrascan CCD 2k–2k and digital diffractograms were calculated using the Gatan Digital Micrograph program. Moreover, to be representative and statistically meaningful, many images from several regions of various samples were recorded and the most characteristic results are presented here, and at least 300 NPs were treated. Powder X-ray diffraction was used to determine the phase composition and crystallite size of TNs and was performed with a Philips PW1820 diffractometer.

### Sarcoma cell lines and culture conditions

Sarcoma cell lines, named IB106 and IB115, were characterized and obtained from Institut Bergonié (Bordeaux, France)^83^. The two cell lines were maintained in RPMI 1640 GlutaMAX (Thermo Fischer Scientific, Illkirch, France, Cat. No. 61870036) supplemented with Fetal Bovine Serum (10% v/v, FBS, Thermo Fischer Scientific, Illkirch, France, Cat. No. 16170-078) and streptomycin/penicillin (100 µg mL^−1^, Thermo Fischer Scientific, Illkirch, France, Cat. No. 15140-122). Cells were kept in a humidified atmosphere at 37 °C and 5% (v/v) CO_2_. For both electron and proton irradiation experiments, 20,000 cells were seeded in a drop in specific cell dishes 20 h before irradiation. As a control, a mock sample was used, which was treated in the same way except for irradiation.

### Cell culture, titanate-scrolled nanosheets, and irradiation exposures

Sarcoma cell lines were grown in the defined medium at 37 °C in a 5% (v/v) CO_2_, humidified atmosphere, and passages were realized at 80% confluency. The suspensions of TNs were prepared in ultrapure water at a concentration of 1 mg mL^−1^. TNs were dispersed by intense sonication pulses of 1 min at RT (750 W, 20 kHz, with 30% amplitude) using a dedicated 3MM conical microprobe with Vibra-Cell™ (750 W, 28% amplitude, Sonics & Materials, Inc., Newton, CT, USA). Suspensions were hereby known as “stock suspensions”. Stock suspensions were diluted at the appropriate concentration in a defined culture medium to obtain an exposure suspension at 2 µg.cm^−2^ (final concentration). Briefly, 20,000 cells were seeded in a single drop in the middle of a 6-well plate for 24 h in an appropriate culture medium, and then exposed to TNs for 16 to 24 h. Cell irradiation was carried out at the Department of Radiotherapy, Institut Bergonié (Bordeaux, France) using a Clinical Linear Accelerator (CLINAC 21EX, Varian Medical Systems) used in the routine treatment of patients. Sarcoma cell lines were irradiated using 6 MV photon beams with 1, 2, and 4 Gy delivered at a dose rate of 2 Gy.min^−1^. These doses are selected as irradiation doses because 2 Gy is the fractionated dose used during patient treatments; 1 and 4 Gy are lower and higher doses compared to this standard value. Source Fantom Distance (SFD) of 100 cm was applied at the surface of a certain amount of equivalent water 30 × 30 cm^2^ slabs (RW3, PTW) providing sufficient backscatter conditions (> 15 cm) for a 6 MV photon beam (TRS 398, IAEA). The cell monolayer was covered by a total of 15 mm of water equivalent medium to achieve electronic equilibrium (10 mm of water in the wells and 5 mm of plastic slabs above). Interstices were also filled with water to prevent the creation of heterogeneities that could disturb the secondary particle fluences and hence alter the dose deposition precision. The cell monolayer was covered by 10 mm of medium to achieve electronic equilibrium. To maintain this depth without removing the TNs, 9.6 ml of the growth medium was added one hour before irradiation. The cell monolayer was covered by a total of 15 mm of water equivalent medium to achieve electronic equilibrium (10 mm of water in the wells and 5 mm of plastic slabs above). Interstices were also filled with water to prevent the formation of heterogeneities that could disturb the secondary particle fluences, and hence alter the dose deposition precision. The photon beam was collimated in a 15 × 15 cm^2^ square field at isocenter and irradiations were carried out with a single beam oriented at 0° (single vertical beam). The dose verification was performed on the day of the experiment as it is performed every day before clinical treatment, and the criterion of acceptability is that the variation in dose is less than 1% compared with the local reference. As a control, a mock sample was used, which was treated in the same way except for TNs exposure and/or irradiation.

### Cellular proliferation assay

Sarcoma cells were harvested with trypsin–EDTA (0.05%, v/v, Thermo Fischer Scientific, Illkirch, France, Cat. No. 25300062) right away irradiation, and 2000 cells were seeded in a 12-well cell culture plate (Cellstar™, Grenier Bio-one, Dutscher Sas, Bernolsheim, France, Cat. No. 665180). Every 2-day the culture medium was removed, cells were paraformaldehyde fixed (4% w/v, Sigma-Aldrich, Saint-Quentin Fallavier, France, Cat. No. P6148-500G) in phosphate-buffered saline (PBS 1X, pH 7.4, Thermo Fischer Scientific, Illkirch, France, Cat. No. 10010023) for 15 min at room temperature and conserved at − 20 °C in ethanol 70% (v/v). Cell nuclei were stained for 10 min with Hoechst^33342^ (1 µM, Thermo Fischer Scientific, Illkirch, France, Cat. No. H3570). Data acquisitions were performed with a Zeiss AxioObserver Z1 microscope (Carl Zeiss Micro-Imaging S.A.S, Rueil-Malmaison, France, AxioObserver Z1). Two independent experiments were performed in duplicates.

### Cell preparation for ion beam micro-analysis (IBA)

Sarcoma cells were cultured directly onto ion beam microprobe sample holders as adapted from previous studies^85,86^. Briefly, cells were directly grown on 2 µm-thick polycarbonate foil for 24 h in an appropriate culture medium and then exposed (or not) to TNs for 16 to 24 h before irradiation. Control cells were prepared similarly with no addition of TNs and no irradiation exposure. 24h after the irradiation sequence, cells were rinsed once in the culture medium and very briefly rinsed twice in ultrapure water to remove excess extracellular salts from the culture medium. Finally, cells were plunge-freeze at -150°C into liquid nitrogen chilled 2-methyl butane (ReagentPlus, ≥ 99%, Sigma-Aldrich) and freeze-dried using a freeze-dryer (*Christ alpha*, Thermo Fischer Scientific, Illkirch, France) in two phases: A phase of primary desiccation of 12 to 24 h (T°C = −99 °C, P = 0.001 mbar) followed by a phase of secondary desiccation of 24 h (T°C =  + 40 °C, P = 0.001 mbar). With no contamination and no ionic diffusion, freeze-drying was found to be the most adapted process. Single-cell quantitative analysis of the chemical element (quantitative analysis and element distribution) was carried out with a high-resolution microprobe beamline (AIFIRA, LP2iB) using complementary ion beam analytical techniques (μ-STIM, μ-PIXE). Protocols are described in previous works^84,86^. Micro-PIXE involves the collision of incident protons with target atoms, resulting in the emission of X-rays. This technique provides valuable information about the chemical composition and two-dimensional elemental maps of the sample. It offers numerous advantages, including simple sample preparation, a large observable area with zooming capabilities, easy quantification of chemical elements expressed in ng.cm^−2^, and the ability to extract single-cell content within a cell population. It enables the simultaneous collection of trace elements from sodium to heavy metals, offering sub-cellular spatial resolution and high sensitivity.

### Conversion of calcium content expressed in ng cm^−2^ into µM content

Indirect quantification of calcium content expressed in mole per liter is possible provided the hydrated cellular mass. Then, considering a water content in cultured cells of 97% and a dry cellular mass of 100 µg per square centimeter, the basal content of calcium in control cells, measured around 20 ng.cm^−2^ corresponds to a total intracellular calcium concentration of 5.4 µM.

### Library preparation and sequencing (Oxford Nanopore, PCR-cDNA barcoding).

Two key considerations emerged regarding the experimental conditions. First, we encountered a multiplicity of conditions involving two cell lines, TN exposure at doses of 0 and 2µg cm^−2^, and exposure to IR at doses of 0, 1, and 2 Gy. Second, there is a limitation in the number of samples available for each condition. To address these challenges, we chose to use the Oxford Nanopore Technologies protocol, which allows for long-read and multiplexed sequencing of cDNA collections. The extracted transcriptomes were then prepared for sequencing using the PCR cDNA barcoding kit to sequence multiple conditions on a single chip. The biological triplicates were performed on three different Flow cells.

Total RNA was extracted from cells at different times (from 1 to 24 h) after exposure to NPs and/or irradiation using the RNeasy mini kit (Qiagen SAS, Courtaboeuf, France cat. n^o^ 74104) according to the manufacturer’s instructions. Whole-transcriptome cDNA libraries were first constructed from extracted mRNA using a PCR-cDNA barcoding kit (Oxford Nanopore Technologies, Oxford, UK, cat. n^o^ SQK-PCB109). Briefly, 50 ng total RNA (~ 1 ng (polyA)^+^ mRNA) from each condition was taken in RNase-free PCR tubes containing Maxima H Minus reverse transcriptase enzyme (Thermo Fischer Scientific, Illkirch, France, Cat. No. EP0751), VN primer (2 µM, variant of oligo (dT) with complementary nucleotides for annealing of barcode primers), 10 mM dNTPs (New England Biolabs, Evry, France Cat. No. N0447S), RNaseOUT (40 U/µl, Thermo Fischer Scientific, Illkirch, France, Cat. No. 10777019) and strand-switching primer (10 µM, Oxford Nanopore Technologies Oxford, UK). Products were sequentially added and prepared as recommended by the manufacturers. Samples were incubated at + 42 °C for 90 min followed by enzyme inactivation at + 85 °C for 5 min (one cycle) in a thermocycler for the generation of full-length cDNA from poly(A)^+^ messenger RNA.

cDNA libraries from each sample then underwent full-length amplification and sample barcoding using 14 cycles of PCR. Each PCR reaction mix consisted of 25 µl 2 × LongAmp Taq master mix (New England Biolabs, Evry, France Cat. No. M0287S), 1.5 µl barcoded primers (named BP01 to BP12), 18.5 µl nuclease-free water, and 5 µl (~ 0.25 ng) cDNA. The following PCR cycling conditions were used: initial denaturation at 95 °C for 30 s (1 cycle), denaturation at 95 °C for 15 s (14 cycles), annealing at 62 °C for 15 s (14 cycles), extension at 65°C for 15 s (14 cycles), and final extension at 65 °C for 6 min (1 cycle) (the long extension time of 6 min was to selectively amplify cDNAs up to ~ 5 kb in length). 1 µl of Exonuclease 1 (New England Biolabs, Evry, France, Cat. No. M0293S) was finally added in each PCR tube for incubation at + 37 °C for 15 min and followed by enzyme inactivation at + 80 °C for 5 min (one cycle). To clean up the cDNA libraries, PCR reactions with the same barcode (from BP01 to BP12) were pooled in two 1.5 ml DNA tubes and primer dimers, removed using 0.8 × volume equivalent Agencourt^®^ AMPure^®^ XP beads (Beckman Coulter, Villepinte, France, Cat. No. A63880). Briefly, beads (80 µl) were added to each pooled sample, incubated on a hula mixer for 5 min at room temperature, and spun and pelleted on a magnet. Supernatants were pipetted off and the resulting beads were washed with 70% (v/v) ethanol (200 µl freshly prepared using nuclease-free water) without disturbing the pellet. The ethanol was removed using a pipette and the beads were washed again with ethanol, and the pelleted beads spun down and placed back on the magnet. Residual ethanol was pipetted off and the beads were briefly allowed to dry. While the beads still appeared glossy (with no cracking) they were resuspended in 12 µl elution buffer (provided with the PCR-cDNA barcoding kit; Oxford Nanopore Technologies, Oxford, UK) to recover the cDNA libraries. Before loading the barcoded cDNA libraries onto the flow cell for long-read sequencing, 100 ng of each cleaned-up cDNA library were ligated with adapters. The barcoded libraries (100 fmol final) were pooled in a 1.5 ml DNA, combined with Rapid adapters (Oxford Nanopore Technologies, Oxford, UK, Cat. No. SQK-PCB109), and incubated at room temperature for 5 min. The adapter-ligated pooled cDNA library was transferred to a fresh tube and combined with loading beads and sequencing buffer (SQK-PCB109 kit) to form the sequencing mix. Three flow cells R9.4.1 (Oxford Nanopore Technologies, Oxford, UK, Cat. No. FLO-MIN106D) were primed with pre-mixed flush buffer and flush tether (Oxford Nanopore Technologies, Oxford, UK, cat. no. Flow Cell Priming kit EXP-FLP002) then loaded with the sequencing mixes and run for 24 h on a MinION Mk1C sequencing device (Oxford Nanopore Technologies, Oxford, UK). The sequencing libraries from one of the IB115 triplicates were of lower quality than the other libraries, resulting in fewer reads of poorer quality. It was therefore decided to exclude this IB115 triplicate from the rest of the analysis to avoid introducing a *bias* linked to sample preparation.

### Transcriptome analysis

Upon completion, *guppy v6.1.4* was used to perform high-accuracy base-calling and demultiplexing of the data (Raw *fast5* files). Reads were mapped with *minimap2* option '-ax map-ont' to the Homo sapiens GRCh38 transcriptome and pooled into an expression matrix via a dedicated Python script. Differential expression analysis was performed on R with *edgeR* and *limma* packages. Genes with expression intensity lower than 1 count per million (cpm) are considered non-expressed and discarded from the analysis.

#### PCA

Principal Component Analysis was performed with the plotMDS (*limma*) on the unnormalized expression matrix of all genes > 1cpm. The Euclidean distances were obtained from the coordinates position of the 2D representation of PC1 and PC2 values in all experimental conditions and presented via a cluster map (*seaborn* Python package). The top 100 genes with the most weight on PC1 and PC2 respectively were extracted for later comparison with differentially expressed genes.

#### Differential expression

Data are normalized (*limma-voom*) and transformed into log2, a linear model is then adjusted for each gene. The contrasts are then extracted and compared to the linear model according to the empirical Bayesian method, the obtained p values are adjusted by the Bonferroni method^87^ to obtain the final differential expression results.

#### Enrichment analysis

Enrichment analysis is performed with the *gprofiler2*^88^ R package, which queries public APIs from several databases and determines statistically significant results based on the number of impacted genes and the total number of genes attached for each ontology. It is worth noting that we did not differentiate the DE genes based on whether they were under-expressed or over-expressed, as our primary focus was on identifying the types of impacted pathways in the cellular response to the exposure conditions.

### Supplementary Information

Supplementary Information.

## Data Availability

The raw data supporting the conclusions of this article will be made available by the authors, without undue reservation (contact H. Seznec, herve.seznec@cnrs.fr). The datasets generated and analysed during the current study are available in the Gene Expression Omnibus (GEO) repository [(GSE248011) (NCBI tracking system #24377339)].
