# Supplementary material for: Sarcoma cell-specific radiation sensitization by titanate scrolled nanosheets: insights from physicochemical analysis and transcriptomic profiling
Source: Sci Rep. 2024 Feb 8;14:3295. doi: 10.1038/s41598-024-53847-x (PMC10853196; doi:10.1038/s41598-024-53847-x)
Supplement: Supplementary file 1 — Supplementary Information. [file 41598_2024_53847_MOESM1_ESM.docx]

**Sarcoma Cell-specific Radiation Sensitization by Titanate Scrolled Nanosheets: Insights from Physicochemical Analysis and Transcriptomic Profiling**

Pierre Beaudier^1,2‡^, Florent Vilotte^1,4‡^, Marina Simon^1‡^, Giovanna Muggiolu^1‡^, Quentin Le Trequesser^3^, Guillaume Devès^1^, Laurent Plawinski^1^, Antoine Mikael^4^, Jérôme Caron^4^, Guy Kantor^4^, Denis Dupuy^2^, Marie-Hélène Delville^3^, Philippe Barberet^1^ and Hervé Seznec^1*^

*(1) Univ. Bordeaux, CNRS, LP2I Bordeaux, UMR 5797, F-33170 Gradignan, France*

*(2) Univ. Bordeaux, INSERM, IECB, U1212– F-33607 Pessac - France*

*(3) Univ. Bordeaux, CNRS, Bordeaux INP, ICMCB, UMR 5026, F-33608 Pessac, France*

*(4) Radiation Oncology Unit, Institut Bergonié, F-33076 Bordeaux, France*

***^‡^*** *The authors wish it to be known, in their opinion, the first four authors (PB, MS, GM, FV) should be regarded as joint First Authors*

* Corresponding author: Hervé Seznec

**Supplementary data**

**
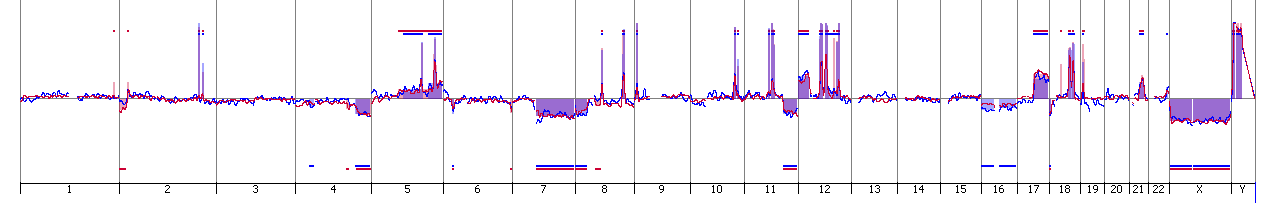
**

**Supplementary Figure S1: Histology, comparative genomic genomic hybridization profile (CGH), and genomic stability of IB115.**

The IB115 cell line originates from a dedifferentiated liposarcoma of a para-testicular tumor, following surgical extraction at, Institut Bergonié. This sarcoma cell line exhibits a straightforward genetic profile, characterized by limited amplifications of the MDM2/CDK4 genes located on chromosome 12q15. For 50 passages, the stability of the IB115 cell line has been rigorously assessed. Remarkably, the CGH profile has remained unchanged, as evidenced by the congruence between the CGH profiles after 30 and 50 passages of culture, and unequivocally demonstrates the remarkable genomic stability of this cell line. The CGH profile showcases the distinctive alterations typically associated with dedifferentiated liposarcomas, featuring amplifications (indicated by upward-pointing vertical peaks) and localized deletions (areas below the midline). Apart from these well-known variations, the overall profile exhibits no significant alterations. Notably, localized amplifications of the MDM2 and CDK4 genes on chromosome 12q15, a hallmark of liposarcomas, are consistently observed. The overlay of CGH profiles for IB115 cells at passages 30 (in blue) and 50 (in red) underscores the considerable genomic stability maintained over time.


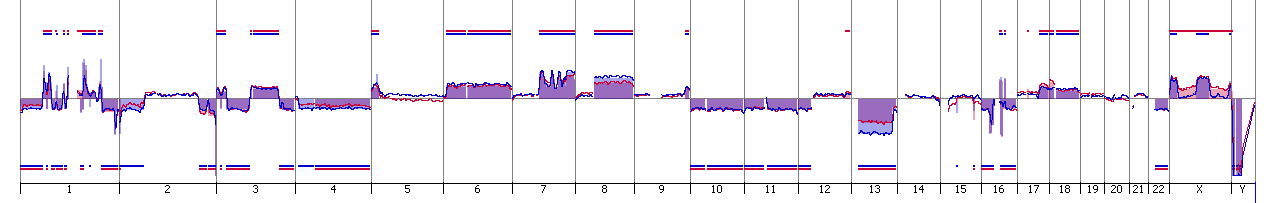


**Supplementary Figure S2: Histology, comparative genomic hybridization profile (CGH), and genomic stability of IB106.**

The IB106 cell line is derived from an unclassified sarcoma found in a paravertebral mass, characterized by a heterogeneous population of pleomorphic cells. This cell line is known for its intricate genetic makeup, and its remarkable genetic stability over time is illustrated by the convergence of the CGH profiles in blue and red, as presented. The profile of IB106 reveals substantial alterations specific to this unclassified sarcoma. In contrast to IB115 cells, this cell line exhibits a greater number of amplifications (evidenced by upward-pointing vertical peaks) and deletions (regions below the midline). These numerous genetic modifications collectively define the complexity of this tumor's genetics. Notably, the CGH profiles obtained at passages 30 and 50 consistently demonstrate the enduring stability of this cell line, despite its intricate genetic landscape.


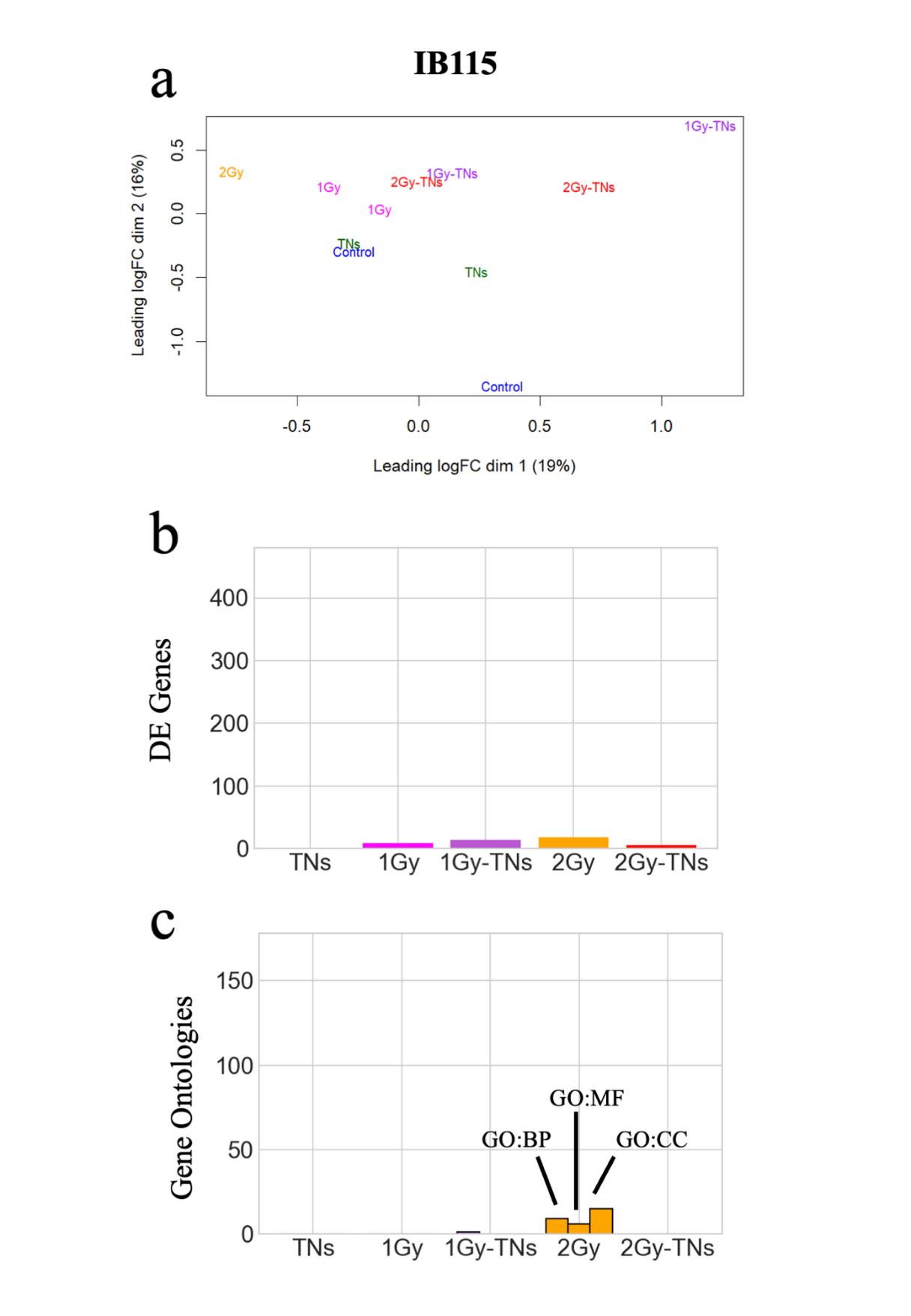


**Supplementary Figure S3.** Differential expression results on all the experimental conditions of the IB115 cell line (a) Principal Component Analysis using all genes >1cpm and non-normalized gene expression matrix, (b) number of DE genes per condition, (c) Number of significantly (*p-value*<0.05) impacted Gene Ontologies obtained using the gprofiler2 R package per experimental condition. The three bars per condition represent respectively a Biological Process, a Molecular Factor, and Cellular component ontologies.


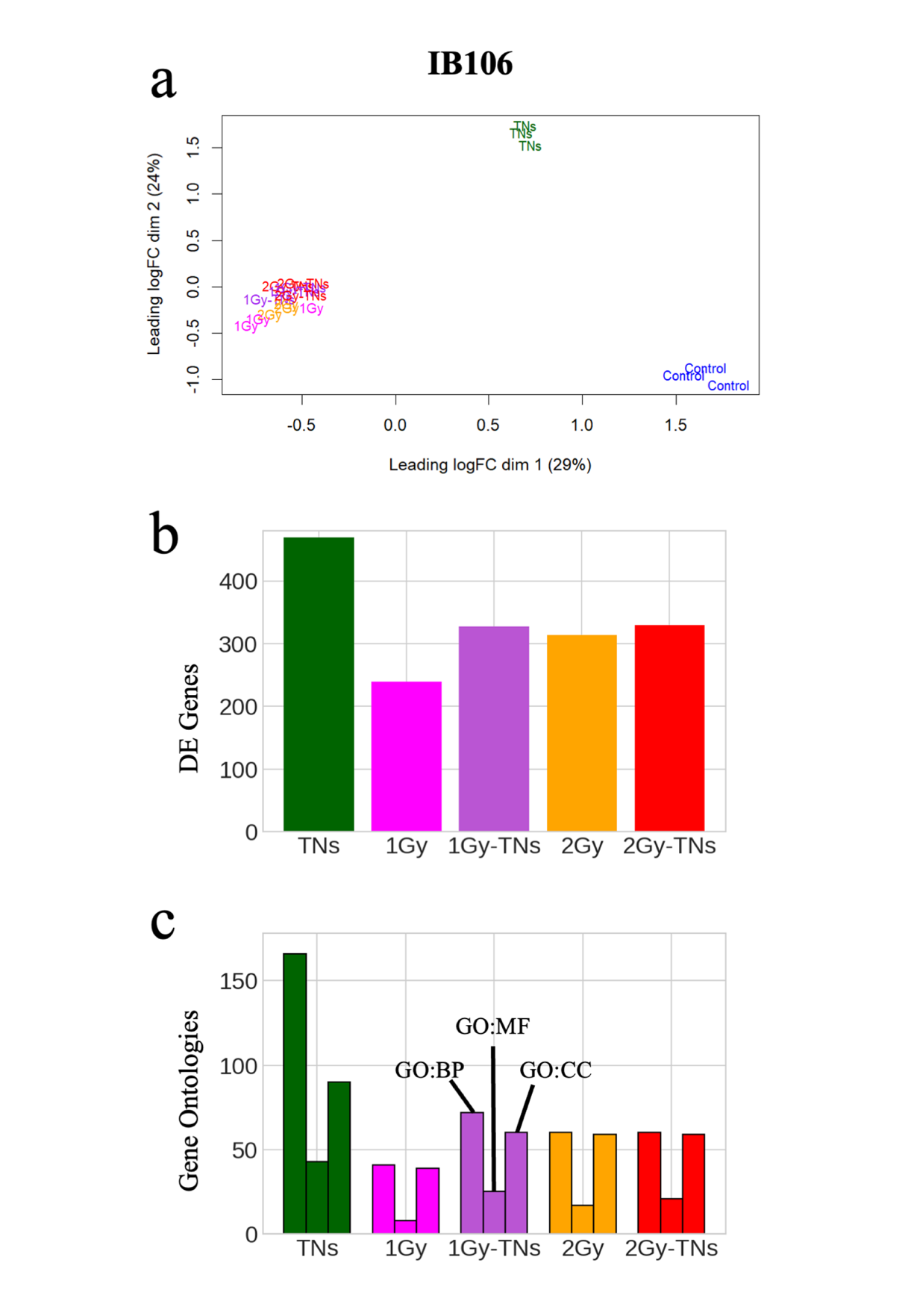


**Supplementary Figure S4.** Differential expression results on all experimental conditions of the IB106 cell line (a) Principal Component Analysis using all genes >1cpm and non-normalized gene expression matrix, (b) number of DE genes per condition, (c) Number of significantly (*p-value*<0.05) impacted Gene Ontologies obtained using the gprofiler2 R package per experimental condition. The three bars per condition represent respectively Biological Processes, Molecular Factors, and Cellular components ontologies.

**Supplementary Table S1.** List of the Differential Expressed genes classified by Genes Ontologies identity number and significantly impacted in the case of IB106 line (adjusted *p-value* < 0.05) and according to the different experimental conditions. List of genes related to the identified metabolic pathways: Protein metabolism.

**Supplementary Table S2.** List of the Differential Expressed genes classified by Genes Ontologies identity number and significantly impacted in the IB106 line (adjusted *p-value* < 0.05) and according to the different experimental conditions. List of genes related to the identified metabolic pathway: Cell cycle.

**Supplementary Table S3.** List of the Differential Expressed genes classified by Genes Ontologies identity number and significantly impacted in the case of the IB106 line (adjusted *p-value* < 0.05) and according to the different experimental conditions. List of genes related to the identified metabolic pathway: Stress/apoptosis.

**Supplementary Table S4.** List of the Differential Expressed genes classified by Genes Ontologies identity number and significantly impacted, in the case of IB106 line (adjusted *p-value* < 0.05) and according to the different experimental conditions. List of genes related to the identified metabolic pathway: Respiration.
